# Tracing Chinese international students’ psychological and academic adjustments in uncertain times: An exploratory case study in the United Kingdom

**DOI:** 10.3389/fpsyg.2022.942227

**Published:** 2022-09-20

**Authors:** Guangxiang Liu, Wentao Li, Yueshan Zhang

**Affiliations:** ^1^Department of English, The Chinese University of Hong Kong, Hong Kong, Hong Kong SAR, China; ^2^Department of Language and Literacy Education, The University of British Columbia, Vancouver, BC, Canada; ^3^School of English, The University of Hong Kong, Hong Kong, Hong Kong SAR, China

**Keywords:** psychological adjustment, academic adjustment, Chinese international students, higher education in the United Kingdom, COVID-19, qualitative case studies

## Abstract

The worldwide spread of COVID-19 has exerted tremendous influences on the wellbeing of international students and the sustainable development of higher education. The current study adopts an 8-month exploratory case study to trace eight Chinese international students’ psychological and academic adjustments in the United Kingdom amid the COVID-19 pandemic. Emerging from the qualitative data constitutive of semi-structured interviews, self-reflection writings, memoing, together with stimulated-recall interviews, findings have demonstrated the three main types of obstruction for such students’ adjustments in the foreign land including COVID-specific challenges (i.e., the threat of infect, reduced access to university facilities and resources); COVID-enhanced challenges (i.e., anxiety exacerbated by parents and social media use, anti-Asian racism and hate incidents); and language barriers and cultural differences as long-standing issues. Students’ previous lockdown experience, individual resilience, development of monocultural friendship patterns, and institutional provision and support are all factors that have contributed to their ability to overcome or at least mitigate the psychological and academic difficulties. The study offers insight into the impacts of COVID-19 on international students, providing implications that could contribute to the sustainable adjustments of international students in times of disruptive events and inform future responses to global health crises from individual and higher education perspectives.

## Introduction

The global surge of COVID-19 has turned out to be one of the severest pandemics and public health crises in the 21st century and has generated deep repercussions on many aspects of our individual life and society, as well as the ways in which we participate in the global structure in times of disruption and uncertainty (Caserotti et al., 2021; He et al., 2021; Khorram-Manesh et al., 2021; Siegrist et al., 2021; Tsoy et al., 2021; Yuan et al., 2021; Moosavi et al., 2022). In a bid to curb and respond to COVID-19, there exist a large number of prevention and control strategies such as travel restriction, vaccination promotion, epidemic surveillance and risk perception, ongoing quarantine and lockdowns, which combine to subject people from all walks of life to the influence of this global pandemic (Caserotti et al., 2021; He et al., 2021; Tsoy et al., 2021).

In the field of British tertiary education, higher education institutions witnessed a sudden decline in the enrollment of international students in 2020 due to the worldwide outbreak of COVID-19 which has decreased international mobility and posed a lasting threat to the physical and mental health of international students who tend to be more vulnerable during the pandemic on account of their temporary immigration status (Firang, 2020; Xu et al., 2021; Mok et al., 2022). In addition, due to travel restrictions, campus closure, the suspension of in-person classes, and the abrupt transition to online learning mode, the COVID-19 pandemic has adversely borne upon these students’ intercultural learning experiences and made serious inroads into the sustainability of higher education that figures in maintaining quality education in unforeseen and disruptive events (Pilotti et al., 2022). Things seemed not to become better even in 2021. With the rampant spread of Delta variant in the United Kingdom, national and regional COVID lockdowns were announced and lifted several times. These emergency-imposed changes put British higher education institutions at the risk of the financial crisis, because many universities rely heavily on international student fees which account for almost half of all university income (Popov and Isard, 2022). Moreover, as British higher education institutions wrestled with lockdowns and strict social distancing requirements, many universities were still thrown into disarrays, and it constituted a principal question to help international students to stay healthy and energetic and develop new pedagogies that fitted well with online and hybrid classes. As such, more support for enrolled international students and actions ascertaining how to help them adjust to life amid COVID-19 are required to sustain their psychological and academic development so that they can commit to seeing their studies through to completion in hard times. In this sense, as Crawford and Cifuentes-Faura (2022) point out, there is an urgent need for ‘critical divergence from the pre-pandemic social missions of universities and higher education institutions’ (p. 2).

Nevertheless, despite the growing body of literature that delineates the ways in which the global COVID-19 pandemic has reshaped individual life trajectories, learning experiences, educational development, and socio-economic and political upheavals (Buheji et al., 2020; Firang, 2020; Gover et al., 2020; Marstaller, 2020; see Bolumole, 2020; Khorram-Manesh et al., 2021; Maican and Cocoradǎ, 2021; Xu et al., 2021; Mok et al., 2022), there is still a scarcity of research on how those students who still choose to pursue higher education degrees abroad struggle to negotiate adjustments as outsiders in a foreign land, especially in face of inconveniences and threats brought about by the pandemic. Equally scant is the in-depth and longitudinal investigation into international students’ psychological and academic challenges amid the pandemic. A holistic understanding of the varying difficulties and coping strategies that they manage to develop is able to contribute to constructing a supportive and inclusive higher education environment that sustains international students’ overseas studies and adjustments in the host society in disruptive times. To these ends, this paper sets out to trace the psychological and academic adjustments achieved by Chinese international students in the United Kingdom in the context of the COVID-19 pandemic with a particular view to gaining insights into their lived experiences in the new living and learning settings. In doing so, the present study may also enrich academic insights into the impacts of COVID-19 on international students, providing empirical evidence that could inform future responses to global health crises.

## Literature review

### Conceptualizing adjustment from an intercultural perspective

A spate of research in the field of intercultural communication has made efforts to explain the process by which individuals become accustomed to a different culture and integrate into the host society (Ward and Kennedy, 1999), but there are still variations as to the ways in which adjustment, adaptation, and acculturation are used to indicate different stages of the process. It is therefore necessary to spell out the differences between these three constructs ahead of engaging in any analysis. In light of Zhu (2016), acculturation is frequently used in anthropology, while adaptation and adjustment are often regarded as biological and psychological terms respectively. Furthermore, Berry (2005) defines acculturation as ‘the dual process of cultural and psychological change that takes place as a result of contact between two or more cultural groups and their individual members’ (p. 698), taking the view that acculturation can occur at both group and individual levels for a protracted period. In contrast, adjustment and adaptation only feature in individual changes. Adaptation tends to be applicable in the case of long-term sojourners (e.g., refugees, doctoral students), denoting their progress in cross-cultural transition (Zhu, 2016). Adjustment, by contrast, refers to the alterations made by short-term residents such as 1-year international postgraduate students as a means of getting used to their new living and social environment, which tends to be subtle and less permanent than adaptation (Quan et al., 2016). Seeing that we focus on the relatively short period of time that Chinese international students spend as transborder and transcultural sojourners in the context of COVID-19, the present study employs the term adjustment to replace the word adaptation to capture their dynamic learning and living states at United Kingdom universities amid the pandemic.

### Psychological and academic adjustment

As aforenoted, adjustment is frequently used in psychological studies to describe the efforts made to balance conflicting needs arising within a new environment (Schachner et al., 2016). Drawing on the idea of Ward and Kennedy (1993) regarding adaptation on the mental and emotional levels, psychological adjustments, in this study, refer to individual psychological satisfaction and wellbeing when staying in a different cultural milieu on a temporary basis. Difficulties that cross-cultural sojourners may encounter in the course of psychologically adjusting to new settings include feelings of disorientation, loneliness, problems relating to their identity and anxiety (Ward, 1996). Existing studies reveal that rich personal and interpersonal resources are significant to ensuring psychological wellbeing in intercultural settings. Specifically, Bochner et al. (1977) discuss the friendship patterns of international students and posit those friendships with co-nationals and host nationals (e.g., academics, fellow students, advisors, and officials) can facilitate adjustments made by international students so as to increase their sense of integration and familiarity to the host culture. In a similar vein, Ward and Kennedy (1993) find that effective interactions with host nationals are positively linked with facilitating general adaption to overseas life, reduced levels of academic and social difficulties, and improved linguistic competence. Moreover, in their investigation of two cohorts of college Chinese students in the United Kingdom, Spencer-Oatey and Xiong (2006) demonstrate that intergroup relations and active interactions with foreign students correlate positively with the ability to successfully adjust to daily life. On this ground, Evans and Morrison (2011) further add that a strong command of the target language or local dialects can help international students overcome language barriers and promote their psychological adjustments.

Apart from these factors that can make contributions to the psychological adjustments of international students, scholars have also highlighted the role of self-efficacy and social support in such processes. In the investigation of Korean international students in Australia, Mak and Kim (2011) verify that self-efficacy can have a vital bearing on such students’ psychological and academic adaptation, with those with a low level of self-efficacy being more likely to demonstrate signs of maladjustments. Yu et al. (2019) report social support as another potential dimension in successful psychological adjustments, and state that students with adequate social support are less likely to experience depression and more likely to feel socially connected. These empirical studies indicate that personal and intergroup resources are conducive to helping students engage in positive mental adjustments within the context of the host culture.

Despite the significance of psychological adjustments, a considerable number of studies have argued that study-related problems constitute the primary source of concern among international students (Zhou and Todman, 2009; Yu, 2010). In this sense, academic adjustments can be drawn upon to encapsulate the process of adjusting to a different environment in relation to the matter of learning. Tinto (1993) construes this as the competence to achieve positive educational performance in the host country. However, beyond only academic progress, academic adjustments also entail gaining an understanding of the rules that govern interpersonal relations in the host country, with even seemingly capable students often leading lonely and isolated lives (Klein et al., 1971). This is supported by Gu et al. (2010) who argue that academic adjustment involves a complex set of shifting associations between ‘language mastery, social interaction, personal development, and academic outcomes’ (p. 20).

The experiences of international students’ academic adjustments have been explored at length in the existing research. Since the boundary between psychological and academic adjustments of international students is not clear-cut and they are, as it were, interconnected in many respects (Zhu, 2016), some factors that figure in influencing students’ psychological issues also take a role in mediating their academic adjustments. Thus, one consensus has been reached that English language proficiency, individual attitudes or experiences, and host culture support are all crucial in facilitating positive academic adjustments. For instance, based on a sample of 359 international students in Europe and Asia, Yeh and Inose (2003) claim that language fluency, support from the host culture and social connectedness all have a significant impact on students’ overseas learning experiences. Yu (2010) examines the academic adaptations of 215 foreign students in China and finds that integrative motivation, language anxiety, and sociocultural adaptation are positively correlated to intercultural sojourners’ academic adjustments. Leong (2015) adopts a qualitative research design to investigate the academic experiences of 11 international students at a single American university and reports that English language competence serves as the primary challenge on the way to academic success. Pedagogically, researchers have also examined the negative academic performance exhibited by certain Chinese international students at universities in English-speaking countries, isolating other potential factors as relating primarily to matters of personality and prior intercultural experiences (Arambewela and Hall, 2013), academic cultural differences (Zhou and Todman, 2009), and insufficient support offered by the host university (Bamford, 2008).

Although these combine to map out the terrains of international students’ psychological and academic adjustments and dissect their multiple contributing factors in pre-pandemic contexts, it remains uncertain about how the COVID-19 pandemic attenuates or complicates such issues, or even generates emergent insights into the adjustments of international students to the host culture and educational environments.

### Chinese international students in times of pandemic

The sojourning experiences of Chinese international students have been a topic of interest in academia. Prior to the pandemic, of relevance is the springing up of research clusters about these students, concentrating on issues such as their intercultural exchanges, international learning experience (Gu and Maley, 2008; Zhu, 2016); the adjustments in varying university contexts (Quan et al., 2016); acculturation trajectories (Zheng et al., 2004); social-cultural and psychological adaptations (Spencer-Oatey and Xiong, 2006); and challenges and identity shifts they encountered and underwent in the host culture (Gu and Schweisfurth, 2015). The COVID-19 pandemic has sparked off a continuation of scholarship connecting Chinese international students with issues regarding mental health and depression, usually situated within the United States context based on large-sample quantitative analysis (Chi et al., 2020; Lai et al., 2020; Wang, 2020; Xia and Duan, 2020; Ma and Miller, 2021; Su et al., 2021; Lin et al., 2022). These studies provide immediate and useful information about the psychological and mental states of Chinese international students, highlighting that as international sojourners in the host society in disruptive times, they are not only subject to long-lasting stress and fear but symbolic violence from the online world such as being stigmatized and labeled as bat-eating Chinese (Wang, 2020; Ma and Miller, 2021). Realizing the deficiency of these cross-sectional survey studies, some of the ensuing studies begin to move beyond web-based questionnaires and attach importance to qualitative design to unearth and interpret the segmented lived experiences of international students in detail. For instance, by narrowing down their focus to the information-obtaining and disseminating practices in an online community dominated by Chinese international students in South Korea, Jang and Choi (2020) argue that such a virtual hub can act as a source of mutual support by enabling the members to comfort each other and build solidarity to cope with the challenging situations. In so doing, this study has reiterated the role of community and social support in sustaining and empowering international students as both linguistic and cultural minorities in uncertain and frightening times. Seeking to expose the real-life experiences of Chinese international students in the United States, Xu et al. (2021) leverage a descriptive phenomenological method to interview 14 target participants, and their findings reveal four transformed meaning units, of which safety concerns and unanticipated support stand out in the phenomenal experiences of these international students. Following up on their preceding quantitative statistical analysis (Lai et al., 2020), Lai et al. (2021) further group-interviewed 20 Hong Kong Chinese international students in western contexts and underlined the central role that cultural differences and cognitive appraisal played in shaping international students’ perceived severity and vulnerability in the COVID lockdown.

However, noteworthy is that these studies tend to be the products of the quick actions taken by researchers and educators, and the majority of them were conducted at the initial stage of COVID-19. To produce insights into facilitating the sustainable development of higher education from the international student perspective necessitates a more prolonged engagement with this group of students. Moreover, the existing literature has touched on the wellbeing of Chinese international students across different contexts (e.g., United States, South Korea), yet little is known about the adjustment situations of Chinese students in the United Kingdom where it has still come to be the second-largest destination for international students in the world. More importantly, it is imperative to fathom these students’ responses to various stressors and whether their coping strategies are effective to provide references for institutions and future international students in the post-pandemic era. To fill in these gaps, the present study is structured to answer the following questions:

**RQ1**: What are the challenges encountered by Chinese international students in relation to achieving psychological and academic adjustments during COVID-19 in the United Kingdom?
**RQ2**: What are the coping strategies that have helped Chinese international students adjust to the new living and learning environment associated with COVID-19 in the United Kingdom?

### Methodology

A longitudinal exploratory case study method was adopted to obtain rich and in-depth information from the daily stories and academic transition experiences of Chinese international students in the United Kingdom amid COVID-19. As Merriam and Tisdell (2016) explain, case study research is beneficial in exploring a phenomenon inseparable from its social context. This can be achieved through an in-depth description and analysis of a small number of “bounded” phenomena in question—or a case. Given that any kind of macro and micro phenomenon being studied can be regarded as a case (Merriam and Tisdell, 2016), the case in this article is the focused Chinese international students in the United Kingdom during COVID-19. Also, the case study also allows the researcher to acquire contextual and concrete knowledge about the subjects, which is thought to be compatible with the current study that sets out to construe the characteristics and meanings made by the case.

### Participants

Given that the study is concerned with the short-term (no more than 1 year) adjustment experiences of Chinese students at United Kingdom universities during COVID-19, the sampling criteria were formulated such that (1) the participants were Chinese students enrolled in the 1-year Master’s programme in the United Kingdom; (2) they were enrolled for the 2020/2021 academic year across a range of different programmes. These standards allowed the present study to account for the heterogenous backgrounds of the participants across the range of programmes that they were undertaking. We initially reached out to those of our social contacts studying at universities in the United Kingdom and asked for their consent to participate in the study. After several rounds of interactions with potential participants via email, eight qualified participants were eventually recruited through purposive sampling These eight participants fulfilled the sampling criteria and did not meet any exclusion criteria of the present study which refer to (1) the potential participants who meet the sampling criteria but present with characteristics that could interfere with the success of the study; (2) the participants were mentally vulnerable and were not suitable for in-depth interviews or the participants were under great pressure from heavy study loads and were not likely to see it through. To avoid any potential harm and inconvenience to the focused cases, importantly, ongoing consent was ensured so that they could choose to withdraw from this research at any time if they feel inappropriate or unwilling to continue to participate in this study.

Among eight qualified postgraduate participants (three men and five women, with an age range from 19 to 27), three of them were studying in England, and three in Scotland, coupled with the rest two in Wales and Northern Ireland respectively (see Table 1). As of the time we established contact (i.e., November 2020), all participants had lived in the United Kingdom for at least 2 months and had undergone the arrival quarantine and several rounds of COVID-19.

**TABLE 1:** Participant profiles.

Participant	Gender	Field of study	Postgraduate (PG) or undergraduate (UG)	Region
1	Female	Education	PG	Scotland
2	Female	Education	PG	Scotland
3	Female	Public Relations	PG	Scotland
4	Female	Fine Arts	PG	England
5	Male	Economics	PG	Wales
6	Male	Translation	PG	England
7	Female	Photography	PG	England
8	Male	Environmental Science	PG	Northern Ireland

### Data collection and instruments

**FIGURE 1:**
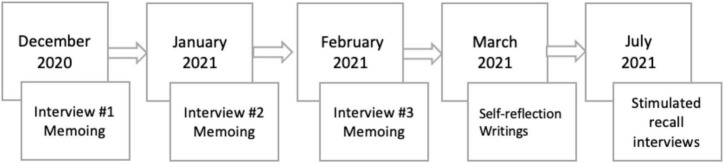
Data collection timeline.

Data collection lasted from December 2020 to July 2021. Prior to data collection, we engaged in communication with each participant to establish rapport and elicit their stories and narrations. Later, we gathered data via semi-structured interviews with participants, their self-reflection writings, researchers’ memoing, as well as stimulated recall interviews (see Figure 1).

#### Semi-structured interviews

The semi-structured interviews were organized based on the three-phase interview model outlined by Seidman (1998) to acquire thorough and in-depth information (see Supplementary Appendix I). In light of Seidman (1998), in-depth and phenomenological interviewing features ‘conducting a series of three separate interviews with each participant’ to plumb complex issues in educational studies (p. 16), because it is less likely to explore the topic in detail via solely one-shot meeting with the person whom the researchers have never met. Therefore, getting important insights into the issues in qualitative research demands the three-interview series, with the first aiming to establish the context of participants’ experience and the second construing the details of participants’ experience within the afore-constructed context, in conjunction with the last allowing participants to reflect on the meaning of their experiences (Seidman, 1998). Building upon this model, all participants in this study were individually interviewed three times in Chinese via Zoom over a time span of at least fourteen days (between December 2020 and February 2021). The purpose of the first interview was to enquire into participants’ past learning and level of preparedness before moving to the United Kingdom (see Table 2). Thus, all interviewees during this stage were asked about their personal backgrounds and past experiences in China during COVID-19. Drawing upon the study of Spencer-Oatey and Xiong (2006) which investigated Chinese students’ psychological and sociocultural adjustments to the United Kingdom, the second interview was structured into three sections, daily life, social life, along with academic studies. The interviewees were asked questions such as their experiences of daily life, psychological and academic difficulties, and their coping strategies. In a bid to further interpret the meaning that participants constructed, the third round of interviews encouraged participants to reflect on previous interviews and probe for any additional information that helps to answer the two research questions. All interviews were audio-recorded and stored in online encrypted files.

**TABLE 2:** Phases and topics of semi-structured interviews.

Interview	Focus	Topics	Average time length
1	Life trajectories of participants	Past learning and living experiences in China	40 mins
2	Ongoing experiences in lockdown	Details of their adjustment process	60 mins
3	Exploring additional information in a relaxed atmosphere	Reflection on the previous two interviews	30 mins

#### Self-reflection writings

After being interviewed three times, each participant was asked to provide a written form of self-reflection on the basis of the information they had supplied during their interviews to triangulate the data sources and reduce researcher bias. These reflections were allowed to be written either in English or Chinese and were not constrained by a word limit (see Supplementary Appendix II for details).

#### Memoing

Memoing (or the researchers’ reflective notes) constituted the third source of research data. Groenewald (2004) emphasizes the value of memoing in qualitative research methods for its contribution to reflections on the data-collection process itself. Field notes were kept amidst each interview, and memoing transpired in the course of compiling and reflecting on these notes.

#### Stimulated recall interviews

Differing from previous cross-sectional research, this study seeks to trace the psychological and academic adjustments of Chinese postgraduate students in the United Kingdom, rather than being a one-off exploration. In this respect, stimulated recall interviews were drawn upon as a technique to locate the focus students’ changes over a relatively extended period of time, so as to shed a light on why informants act and respond in certain ways as shaped by their experiences in various situations through instigating more in-depth dialogs and reflections (Dempsey, 2010). Specifically, through playing them audio recordings of their previous interviews and summarizing their self-reflection writings, we guided them to retrospectively engage in the interactions to generate answers of “I did” instead of “I might have” (Dempsey, 2010, p. 350). Also noted is that building upon the interview protocols (see Supplementary Appendix I), we asked them to re-answer some questions intermittently. The elicited information would be constantly compared and contrasted to their previous interview results to better capture their dynamic or fluid adjustment processes over the past months.

### Data analysis

Data analysis was an ongoing process that occurred over the course of the data collection and report generation. It should be noted that all interviews were conducted in Chinese to better elicit the stories and experiences of the focused Chinese students. Also, given that all participants were required to write their self-reflection in the language that they preferred, the majority of self-reflection writings were in Chinese. Thus, the very first step is data transcription, and all interviews were transcribed verbatim in English and saved in the form of Microsoft Word files together with the filed notes and the transcribed participants’ reflection writings. Importantly, when transcribing these qualitative data, we paid special attention to the achievement of interrater reliability. To this end, all interviews and self-reflection writings were transcribed by the three researchers respectively. The three versions of transcribed data were compared and constated constantly to ensure the reliability and credibility of these data.

In the following stage, thematic analysis, as defined by Braun and Clarke (2006), was used to identify, analyze, organize, describe, and report themes emerging from the large data set. Specifically, after familiarizing ourselves with the data, we imported all textual data into the qualitative data analysis software NVivo and began coding meaningful textual segments to produce thematic discussions. Subsequently, all relevant codes were sorted and collated for incorporation into the initial themes. By way of illustration, codes including threat of infection and reduced access to university facilities and resources were combined to form the initial theme of COVID-specific challenges (see Supplementary Appendix III for details about the coding scheme). These newly acquired themes were repeatedly refined and reviewed to ensure accuracy and consistency, with the thematic map being repeatedly checked to establish robust and uniform themes. Finally, each theme was carefully named and defined such that the written report could be produced with a final thematic map that presented visually the relationships judged as holding between the different themes.”

## Results and discussion

**FIGURE 2:**
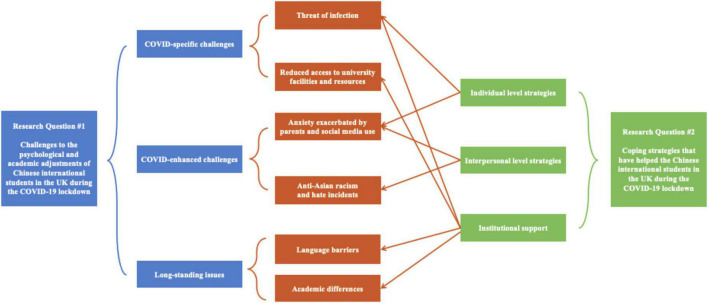
Thematic map of the research question.

The findings of this study suggest that the Chinese international students consulted have generally succeeded in adjusting to life and academic study in their 1-year sojourning period in the United Kingdom against the backdrop of the pandemic. However, it is also the case that, within the data, certain obstacles emerge regarding the psychological and academic adjustments they have had to make. These can be categorized as relating to (1) COVID-specific challenges including the threat of infection, reduced access to university facilities and resources; (2) COVID-enhanced challenges constitutive of anxiety exacerbated by parents and social media, and anti-Asian racism and hate incidents; (3) long-standing obstacles that comprise language barriers and academic differences. As shown in Figure 2, to cope with these difficulties, the Chinese international students have optimized their individual and interpersonal strategies, together with institutional support. The following section presents these results and discusses them in detail.

**RQ1: Challenges to the psychological and academic adjustments of Chinese international students in the United Kingdom during COVID-19.**

### COVID-specific factors

Distinct from previous intercultural studies, it is noteworthy that, the adjusting experiences of intercultural sojourners in this study were largely mediated by the special reality of COVID-19. The fear of getting infected and the reduced access to university facilities and resources became two COVID-specific factors that inhabited Chinese international students’ psychological and academic adjustments during this very time. To start with, the fear of infection was reported by all participants as a primary challenge in psychologically adjusting to the new environment. The majority of participants believed that this fear stemmed from the unsatisfactory response to the crisis on the part of the United Kingdom government(s). This view is evidenced in the interview with Participant 3, who hailed from Wuhan where COVID-19 initially raged and had thus experienced the strictest lockdown in China:

University and government staff barely checked whether newcomers strictly followed quarantine rules and it was all up to you. The lockdown rules were poorly enforced. Just like everybody knew these requirements, only a few abided by them. That’s why I don’t feel safe here and think the United Kingdom government’s response to FF. (Participant 3, semi-structured interview #1)

Similarly, as indicated in other studies concentrating on Chinese international students (Lai et al., 2021; Ma and Miller, 2021; Xu et al., 2021; Lin et al., 2022), most of them were dissatisfied with the host government’s pandemic-curbing actions, condemning the government for the excessive focus on protecting the economy instead of the health and safety of individuals. In the United Kingdom, the rampant spread of COVID-19 mutant strain and the ongoing lockdown in 2021 suggested that the government failed to mount an adequate response, with all British residents still facing the threat of the global pandemic. Therefore, it seems reasonable that all participants took the view that the threat of infection constituted an important source of worry.

Apart from the less reassuring performance of the host government, insecure accommodation environments were also raised as putting Chinese international students at risk, especially in the case of those whose roommates included students with little awareness regarding COVID-19 prevention and low willingness to take protective measures. One participant noted the following:

I have to wear masks everywhere, even in my flat, as one of my native roommates is a real social butterfly and she still parties hard at this very time, and I think it’s extremely risky and dangerous…After 1 month, I had to move out of the apartment to find safe accommodation. (Participant 6, stimulated recall interview)

This finding is consistent with the study of Spencer-Oatey and Xiong (2006) who highlight that the Chinese international students in the United Kingdom featured in their study appeared to feel less acceptable about the noise and the mess associated with foreign roommates with different cultural preferences. In the case of Participant 6, such cultural differences may be embodied in their differing cognitive appraisals of the pandemic severity, which is compatible with the caution of Lai et al. (2021). Also, echoing another relevant study conducted in the inception of COVID-19 (Xu et al., 2021), it further corroborates that safety concerns or the threat of infection, has been the overarching issue that imposes constant pressure on these Chinese students studying abroad.

Reduced access to university facilities and resources was the most significant barrier highlighted regarding integration into academic life. With the advent of the third United Kingdom national lockdown from January 2021 to July 2021, all participants reported that university campuses and libraries had been shut down once again, leaving an adverse impact on students who “felt more productive in libraries than in a self-isolated room” (Participant 1, semi-structured interview #3).

Additionally, the closure of university campuses entailed a hasty transition to online learning, resulting in the unavailability of most institutional facilities and offline resources and thereby making it difficult for some students to adjust to such a new learning mode. One participant raised the following complaint:

I could have taken the computer-assisted translation course in the school computer lab. With the sudden closure of all campuses and libraries, I have to study online and use two tablets at the same time, one for attending the online tutorial, and one for the class practices. It turns out to be ineffective because I easily got distracted every time. (Participants 6, self-reflection writing)

All participants voiced the belief that online learning was also flawed as a result of the diminished social interaction, the overreliance on technology, and the lack of practice-based learning. For example, Participants 7 and 8 were especially emphatic in relation to this point:

I major in Photography, and it is necessary to discuss the photos with the teacher face-to-face, because you know, visual arts are hard to be clearly looked at and fairly assessed on the internet…and I need more real-life interactions with my supervisor, but the COVID turns every teaching event online. Considering the unfavorable online communication environment, sometimes I am not willing to engage in follow-up discussions with him. (Participant 7, semi-structured interview #1)

As an environmental science student, all my field practices have to be canceled or replaced with online presentations…It is a huge disadvantage and ill-advised to study in the United Kingdom amid the pandemic (Participant 8, stimulated recall interview)

As a form of damage to the quality of their international learning, the sudden alteration from physical classrooms to virtual spaces also hindered students’ willingness to interact with their peers, especially for those who preferred face-to-face communications to online discussion behind the screen because “the lack of physical cues often resulted in misunderstandings” and “we were easily going off-topic within a particular thread in online breakout rooms” (Participant 3, self-reflection writing). Exposing the double bind that current international students face, such problems are redolent of the findings of Bolumole (2020), who states that a lack of access to university equipment and facilities can negatively act upon how well students learn through instruction in hard times. This view might be strongly espoused by the students consulted who relied heavily on fieldwork-based learning or need to conduct experiments in laboratories. Taken together, such problems further underscore the requirement for quality control in online learning mode and the must for a sustainable instructional environment in disruptive events where both temporary access to well-planned instruction is guaranteed and the robust learning and teaching ecosystem is recreated (Crawford and Cifuentes-Faura, 2022; Pilotti et al., 2022).

### COVID-enhanced issues

It emerges that seven of the eight participants experienced anxiety and psychological depression as the result of insecure living and social environments. Interestingly, four of the participants expressed that such anxiety was further exacerbated by interactions with their parents and social media:

There was a time I was quite mad at my parents for their excessive and disturbing phoning. They were very overacting to the virus in the United Kingdom and called me ten times a day, which didn’t help at all but got me overly anxious. (Participant 2, self-reflection writing)

Notwithstanding their caring intentions, the parents’ overreactions failed to provide support but engendered a high level of anxiety among their sons and daughters. It is thought-provoking that half of the participants did not rate the role of their parents as highly desirable, possibly because the unfamiliarity of these international student parents with the host society and realities did not enable them to offer effective advice and psychological support that fit the specific situations and individual needs (Xia and Duan, 2020). In addition to unpleasant communications with his family, participant 6 argued that social media was another anxiety amplifier. He found that “regularly checking daily increases in confirmed cases of coronavirus” and “reading the latest COVID-19 map or report on Twitter, Facebook” exacerbated his anxiety. Participant 1 was of the same opinion but added that it was helpful to alleviate this kind of negative feeling through reducing the unnecessary screen time and especially exposure to social media in a workable way. These findings are roughly similar to those of Ward (1996), who states that anxiety is a common problem for new intercultural experiencers when relocating to a new place. However, there is a crucial difference in that Ward reports on anxiety regarded as stemming from culture shock and uncertainty about the future, whereas the students’ anxiety in this paper results from their overacting parents and social media use within the specific pandemic context.

Also, it is found that COVID-19 as a major global crisis not only caused direct effects on the physical and mental safety of the international student group but intensified some socio-political conflicts that further led to some students’ maladjustments in this hard time. This was the case of the surging anti-Asian racism incidents and xenophobic discrimination and violence associated with COVID-19 that begot widespread repercussions on the wellbeing of Chinese international students in the United Kingdom. Six participants reported that they or their Asian friends had encountered varying forms of discrimination ranging from incidents of being spat on, verbal harassment to refusal of service or even physical assault since the outbreak of COVID-19 due to “the sin of bringing Coronavirus to the West” (Participant 3, semi-structured interview #2). As one informant studying in London voiced out:

Once when I went grocery shopping, I was harassed by a teenager who yelled at me and blamed me for the spread of COVID-19. I didn’t get assaulted because I was way taller and stronger than him, but my Singaporean friend was not as lucky as me. He went out alone and was heavily beaten up on street by a group of native people in an unprovoked attack… it is so ridiculous because we did nothing wrong, but we were discriminated against simply because of our skin color and appearance. (Participant 4, semi-structured interview #1)

Notably, anti-Asian racism and hate incidents were not constrained to physical spaces but also in social networks with the use of discriminating and stigmatizing hashtags against Chinese people such as “Chinese virus,” and “Wuhan Virus.” (Participant 7, semi-structured interview #1). These verbal attacks and racial incidents against overseas Chinese people resulted in a lasting sense of insecurity among a part of Chinese international students who, as a result, opted for “only going outside in a group with friends” (Participant 3, semi-structured interview #2) or “staying at home and avoiding unnecessary contact with local people” (Participant 4, self-reflection writing). These findings resonate with symbolic violence that international students from China have encountered across the online and offline spaces, with the accusation of Chinese virus carriers (Wang, 2020). Similarly, more than 106 out of 192 Chinese international students whom Ma and Miller (2021) surveyed articulated that such discrimination could produce serious impacts on their psychological states and reduce their willingness to engage in intercultural communication. In this respect, connecting infectious diseases with group identities should be condemned as it inevitably aggravates mass misunderstandings of this ethnic group and spurs the rise in anti-Asian racism crimes which may be one of the major culprits of damaging Chinese international students’ wellbeing and vitality during the COVID-19 (Gover et al., 2020).

### Long-standing challenges

Apart from these aforementioned COVID-specific and -enhanced obstacles, language barriers and academic differences still stand to be lasting challenges for the adjusting process of Chinese international students in the context of COVID-19. Five out of eight participants reported that they were less willing to interact with teachers and other non-Chinese students due to their limited oral English fluency. Participant 5 shared:

I barely talk to the teacher and other classmates because I know my spoken English is not good enough to express my thoughts, and I am just afraid of being stupid. (Participant 5, self-reflection writing)

Other participants expressed similar concerns regarding their English language competence, especially when discussing academic matters with lecturers and professors, finding it difficult, for example, to “follow the professor who talked very fast and had a thick accent” (Participant 1, semi-structured interview #2), or to follow those whose speech intelligibility was sometimes restricted by the network instability in online tutorials that should have been held in person. This important finding is compatible with other views regarding the role of English proficiency in Chinese international students’ cross-cultural adaptations (Yeh and Inose, 2003; Yu, 2010; Evans and Morrison, 2011; Leong, 2015). As Yu (2010) points out, language competence is an indispensable factor influencing the psychological and academic adjustment of international students, because oral interactions with host nationals and other foreign students are inevitable and count as the most significant parts of their overseas academic lives. Back to the pandemic context, this study also confirms the finding of Xu et al. (2021) that COVID-19 circumscribes the possibilities for these international students to develop English language proficiency through extended self-isolation and reduced human contact, which thus perpetuates the language barrier as a challenge in Chinese international students’ intercultural adjustments.

Academic differences were also reported as negatively affecting all of the participants’ adjustments in their academic lives. The present study highlights three representative problems associated with academic differences: (i) heavy study loads; (ii) slow adaptation to the independent learning style; and (iii) deficiencies in critical thinking. Participant 8 stated the following:

I find it hard to get used to the new learning style… I grew up in the traditional Confucian culture and teachers normally get everything arranged. What I need to do is to finish those prescribed learning tasks, with no need to focus on things outside of homework, but here most students are way more independent and they have clear study plans. (Participant 8, self-reflection writing)

These findings corroborate those of Zhou and Todman (2009) who highlight the difference between Confucian and Western classes and conclude that such differences are likely to manifest in the varying views of the roles of teachers and students, together with differing educational expectations. Also, these results are similar to the study of Li et al. (2018) disclosing that differences between the host academic culture and one’s previous culture play an important role in course readiness and academic satisfaction. In this sense, these findings confirm that language proficiency and academic differences constitute long-standing challenges for Chinese international students regardless of the occurrence of the pandemic.

**RQ2: Coping strategies that have helped the Chinese international students in the United Kingdom during COVID-19.**

Having discussed the challenges encountered by these Chinese international students in adjusting to the effects of COVID-19, this section now elucidates the coping strategies that have helped these same students. Such coping strategies are defined as resources utilized by these students to help them better overcome their COVID-related and other intercultural difficulties. As illustrated in Figure 1, the Chinese international students consulted appear to have relied on individual-level and interpersonal-level strategies and institutional help, with the specific evidence provided by the individual participants indicating that individual-level and interpersonal-level strategies can assist with mitigating COVID-related fears and anxiety, and that institutional support features in addressing both psychological and academic problems.

#### Individual-level strategies

Individual-level strategies emerge as having developed as a result of these participants’ previous lived experience in China and their personal resilience that they took the agency to negotiate in the pandemic setting. For instance, all participants reported that they had experienced the national lockdown in China for at least 1 month before coming to the United Kingdom, with this causing one participant to “fall into the state of disorientation, loneliness, and depression” (Participant 7, self-reflection writing). Fortunately, they all found finally effective ways to address these negative emotions. Such unforgettable experiences became a valuable asset for them amidst acclimatizing to their new lives in the United Kingdom:

I can surely tell you that I had experienced physical and mental distress during the national lockdown (in China), and I had not left my house for a whole month. Just imagine how hard that time is. That’s why I believe life in the United Kingdom couldn’t be worse. (Participant 5, semi-structured interview #2)

Their previous experiences were reported as enabling these students to acquire a higher level of awareness of COVID-19 prevention than their foreign counterparts and to engage in physical and mental preparation before traveling abroad, including “bringing lots of protective clothing, gloves, masks, antiseptic swabs” and “boosting my self-confidence in overcoming difficulties in overseas life” (Participant 1, stimulated recalled interview). Four of the participants also voiced their belief that the optimism and resilience built in their life trajectories played a positive role in coping with psychological and academic difficulties through giving them a sense of self-efficacy. The impact of previous experiences on psychological adjustments reported here is essentially aligned with the prior literature that emphasizes the positive influence of past intercultural experiences on successful subsequent intercultural adaptation (Yu et al., 2019). In relation to the significance of personal resilience, it has been confirmed to be strongly associated with the cultivation of self-efficacy and personal growth which combine to enable international students to maximize the benefits of the internal university learning environment and acquire greater overall satisfaction with their lives overseas either in the pre-pandemic studies (Mak and Kim, 2011; Arambewela and Hall, 2013) or in the research conducted at the early stage of the pandemic (Lai et al., 2021).

### Interpersonal-level strategies

Interpersonal-level strategies emerge from the data set, and it involves international students’ friendship patterns and interpersonal relationships. All participants indicated that they maintained close friendships with co-nationals who shared a similar sense of identity and belongingness, with such individuals able to offer physical help and emotional support:

The first day I arrived in the United Kingdom I was pretty hungry, and it was too late to buy food outside…It was my Chinese roommate who cooked dinner for me, and that made me very moved… I feel strongly connected and being understood by them, and that is something my foreign friends can’t give me. (Participant 2, self-reflection writing)

The majority of participants reported that they had few offline interactions with foreigners due to the fear of infection, and more importantly, the decreased opportunities to interact in person resulting from social distancing requirements. This is evidenced in the experiences of participant 6 who lived with one local student and three international students of other nationalities. He chose to befriend other Chinese students and was reluctant to contact his roommates owing to varying cultural backgrounds and the consideration that some foreigners ignored the warnings and health advice regarding COVID-19 and did not take the coronavirus seriously (Participant 6, memoing). Surprisingly, this pandemic also shifted the participants’ views regarding what it meant to be a successful intercultural communicator from “an open and frank disposition, excellent language competence” to “being risky, … dangerous and not sensible” (Participant 4, semi-structured interview #3). These findings are in line with the friendship model proposed by Bochner et al. (1977) who claim that the primary social networks of overseas students are chiefly monocultural in that students with the same cultural and ethnic values are more likely to cultivate deep friendships and provide each other with emotional support in a transnational environment. Bicultural networks are also of significance in terms of helping intercultural sojourners in adapting to overseas daily and academic lives. (Bochner et al., 1977; Ward and Kennedy, 1993). However, in this study, a monocultural network is more able to benefit international students through rendering them a buffer and a sense of solidarity. This is also the case in Xia and Duan’s (2020) study which elaborates on the outstanding role of Chinese cultural beliefs and identities in relieving Chinese international students’ adjusting stress and shaping their positive experiences during the COVID-19 pandemic. Likewise, as Jang and Choi (2020) reiterate, such a monocultural network enables international students to stay connected and tide them over the crisis in an uncertain age.

### Institutional support

Most of the participants commented that institutional support was of significant benefit to their academic and psychological adjustments amid COVID-19. Seven participants emphasized that there were online support resources for students’ physical and mental health provided by their universities including online psychological counseling, COVID-19 helplines, personal tutor meetings, and COVID-19 hardship funds. Adding up together, these efforts, in no small measure, have allayed the focus students’ negative feelings in face of the new living and learning environment, despite the fact that these various forms of support are not always available due to financial constraints and personnel staffing shortage. Regarding academic support, universities were reported as striving to offer students multiple options and to ensure the quality of online teaching:

All lectures and tutorials were recorded so that students could watch them again. Teachers tried their best to interact with these inactive students online and not to overlook anyone, with great patience and tolerance to students’ mistakes and requests…Such as making the essay deadline more flexible than before…their help is a booster in my academic confidence (participant 1, stimulated recall interview)

Six participants also stated that their universities offered free English writing courses and organized student-led webinars to increase their understanding of the English language and cultures. Although these institutional services could not take every single conceivable need into account, they proved helpful for those student sojourners’ adjusting processes, which has received support from a number of empirical studies. Spencer-Oatey and Xiong (2006), for example, attribute much importance to the facilitative and supportive role that institutional organizations and student unions have in helping students from varying cultures integrate into the host society. Such support from the host culture enables international students to acquire a sense of social connectedness and satisfaction (Zhu, 2016; Yu et al., 2019). In addition, this study aligns with the research conducted by Lai et al. (2021) on social support for international students in times of pandemic, yet there is a slight difference because Lai and her colleagues regard family and friend contact as the major source of such social support, whereas this article underlines the vital role that higher education institutions play in enabling and sustaining international students’ intercultural adjustments amid COVID-19.

### Implications for a sustainable future

Downing Street has announced the removal of domestic legal restrictions against the COVID-19 pandemic in England since February 2022, which may signify the approaching of the post-pandemic era, at least in the United Kingdom, yet of note is that this does not mean the end of the COVID-19 virus but how human beings learn to live with it at minimum cost. Such an end still imposes tremendous challenges to sustaining the wellbeing and intercultural adjustments of future international students in the de-crisis age. As such, the major findings outlined here provide the basis for a number of implications upon which Chinese student sojourners, university authorities, course instructors, and researchers may draw to contribute together to a sustainable future.

On the individual level, students reported in the interview have demonstrated the value of their past experiences that may provide students with empowering resources to help them build resilience and strengthen a sense of self-efficacy. For this purpose, it behooves Chinese international students to take their agency to cultivate positive personal growth through maintaining a hopeful outlook and exploiting setback experiences (e.g., strict national lockdown in China) in confrontation with adversity (Liu and Li, 2021). Equally important is to develop their individualized coping strategies and proactively engage in their monocultural network to share emotional or physical (if feasible) support with each other. In this way, they are able to gain a sense of solidarity that can act as a powerful weapon for them to fight against psychological and academic difficulties in an uncertain era.

On the institutional level, there has been a top priority for institutions to protect international students’ wellbeing, which sets up the foundation of their sustainable intercultural adjustments. This implication is twofold. One is against the backdrop of abandoning all the pandemic restrictions that disregard the danger of contracting the COVID-19 mutants that still lurk in every corner of the campus and city. In this sense, higher education institutions should raise the staff and students’ awareness that a post-pandemic era does not allude to quitting all precautionary measures, and provide a hybrid teaching mode and accessible self-symptomatic testing to minimize the effects of cross-infection. Effective support for those infected international students should also be guaranteed, especially in the case of those clinically vulnerable students. On the other hand, universities should navigate routes to address anti-Asian racism intensified by the pandemic through organizing workshops or other forms of training to deracialize Asian international students on and off campus and empowering them to become the change agents to voice their encounters and combat hatred incidents and ideologies. In addition, it is recommended to promote more effective inter-community and inter-racial dialogs between international students and host students and staff. As aforementioned, this kind of bicultural connection also makes up an important component of the international student sojourners’ intercultural experiences and academic adjustments. Finally, the overarching task for higher education institutions is to control the quality of education in the post-pandemic age, which is also the essential requirement for sustainable education and facilitating international students’ adjusting processes in and out of times of crisis. Toward this objective, it entails institutional efforts and incessant investment in the professional training of teaching and administrative staff in delivering hybrid-mode or offline instruction, along with the development of students’ readiness for such transitions.

Pedagogically, among a series of practical steps is that university instructors ought to improve their understanding of various learning realities of international students, constructing an inclusive classroom environment for students from linguistic and culturally diverse backgrounds. They may also need to consider structuring culturally responsive strategies to take into account international students’ learning needs and styles and to empower them to capitalize on their capital to engage in online or returned offline instruction, in response to the course unreadiness and dissatisfaction with international learning largely shaped by the pandemic.

## Conclusion

With the growing call for attention to the impact of COVID-19 on students studying abroad in international tertiary education institutions, the study has outlined an 8-month exploratory case study to unpack the psychological and academic adjustments of Chinese international students amid the COVID-19 pandemic in the United Kingdom. Through in-depth analysis of eight Chinese international students’ interview data, self-reflection writings, and memoing, this study investigated the individual adjustment experiences of Chinese international students in the United Kingdom in frightening times and uncovered the three main types of obstruction for such students including (1) COVID-specific challenges (i.e., the threat of infect, reduced access to university facilities and resources); (2) COVID-enhanced challenges (i.e., anxiety exacerbated by parents and social media use, anti-Asian racism and hate incidents); (3) language barriers and cultural differences as long-standing issues. These results demonstrated that this global health crisis exerted serious impacts on the safety, health, and wellbeing of Chinese international students in multiple ways. To mitigate these difficulties, Chinese international students in the United Kingdom have leveraged individual-level strategies (e.g., Students’ previous lockdown experience, individual personalities), interpersonal level strategies (e.g., the development of monocultural friendship patterns), and institutional provision and support.

As a longitudinal and qualitative investigation of the experience of Chinese international students during COVID-19, this study is restricted in its small sample and scope. Thus, further research is recommended to examine the wider experiences of Chinese international students, especially those long-term sojourners such as undergraduates, and doctoral students, with a large sample and the focus on issues relating to their adaptations in the pre-pandemic, in-pandemic, and post-pandemic era. Also, more studies should be conducted to ascertain whether Chinese students in other countries report similar difficulties or challenges and whether similar results emerge with international students from other nationality groups. Moreover, given that most student sojourners in this study led a life characterized by isolation and limited opportunities to engage with local society and culture, future studies are recommended to shed light on Chinese international students’ sociocultural adjustment or adaptations in unprecedented times so as to chart the course for the sustainable future. Anyhow, the construction of a sustainable future cannot dispense with the support of continuous academic insights. It should be noted eliminating the adverse influences of COVID-19 on the lived experiences of international students is expected to be chronic and necessitates joint efforts from students, universities, instructors, researchers, along with other stakeholders. We hope that the present study has contributed to solving this problem and would like to see more insights into preparing future Chinese international students for acclimating to the new transborder and transcultural milieu shaped by the worldwide pandemic in times of uncertainty.

## Data availability statement

The raw data supporting the conclusions of this article will be made available upon request.

## Ethics statement

The studies involving human participants were reviewed and approved by the Chinese University of Hong Kong. The patients/participants provided their written informed consent to participate in this study.

## Author contributions

GL contributed to the conception and design of the study and wrote the first draft of the manuscript. WL and YZ helped with data collection and analysis. All authors contributed to manuscript revision, read, and approved the submitted version.
