# Supplementary material for: Tracing Chinese international students’ psychological and academic adjustments in uncertain times: An exploratory case study in the United Kingdom
Source: Front Psychol. 2022 Sep 20;13:942227. doi: 10.3389/fpsyg.2022.942227 (PMC9531010; doi:10.3389/fpsyg.2022.942227)
Supplement: Supplementary file 1 [file Presentation_1.pdf]

## Appendix I: Interview guidelines

There are three separate semi-structured interviews with varying aims in the present study. Depending on which language participants are mostly comfortable with, the questions in each interview were asked in Chinese, English, or a combination of both.

### Interview # 1

The first interview seeks to gain an initial understanding of participants' experience and their previous life trajectories.

**Part 1 - Personal backgrounds**

1. Please introduce yourself regarding your programme, university, and other basic information you would like to mention.
2. How long have you living in the UK?
3. How did you come to the UK? Did you come to the UK along? If not, who did you come here with?
4. Why did you choose to study in the UK during the COVID-19? And what did your parents think about it?
5. Did you make any physical or mental preparation before coming to the UK? If yes, what were they?
6. What do you normally do to relax in your daily life?

**Part 2 - Past experiences of COVID-19 in China**

1. Where did you live or study in China during the COVID-19?
2. How did the COVID-19 in China impact your life and study?
3. To what extent do you think your lockdown experiences in China influence your living and learning in the UK?

### Interview # 2

The second interview is the primary section to understand Chinese international students' daily and academic experiences in the UK during the COVID-19.

**Part 1 - Daily life during the COVID-19 in the UK**

1. Did you experience the quarantine when arriving in the UK? What was like? How do you like it?
2. What is your daily routine during the COVID-19 in the UK? How does the lockdown affect your daily life?
3. Are there any daily issues bothering you at this very moment? How do you response to it?
4. Do you feel safe or insecure when you are living in the UK? Why or why not?
5. What do you think about the UK government and your university's response to the global COVID-19 pandemic?
6. Are you satisfied with your life in the UK? Why or why not?

**Part 2 - Social life during the COVID-19 in the UK**

1. What is your social life like during the COVID-19 in the UK? How does the lockdown influence your social interaction?
2. Do you have many opportunities to physically or virtually interact with local people, people of other nationalities, and your Chinese co-nationals? If yes, how do you feel like the influence of such interactions?
3. Do you stay in touch with your family during the COVID-19 in the UK? What do you think about it?

**Part 3 - Academic studies during the COVID-19 in the UK**

1. How does the COVID-19 in the UK influence your academic studies?
2. What are some challenges and difficulties to your academic studies during the COVID-19 in the UK? How do you deal with them?
3. What has your university manage done to protect the wellbeing of students and facilitate the adjustments of international students during the COVID-19 in the UK? Do you think it is effective? Why or why not?
4. Do you have anything to add regarding your current living and studying situations in the UK?

**Interview #3**

The third interview aims to encourage participants to reflect on previous interviews and probe for any additional information that is helpful to answer the two research questions. there is going to be a time span of two weeks between interview II and interview III so that participants will have plenty of time to think back about previous two interviews.

1. Have you experienced any emerging problems or difficulties for the past two weeks?
2. To what extent do you think you are satisfied with your daily life, social life, academic studies during the COVID-19?
3. Do you think what are the challenges to your adjustments as an international student during this hard time? Why or why not?
4. Do you think what have helped you adapt to your life and academic studies in the UK? Why or why not?
5. Do you have anything else to share?

## Appendix II: Self-reflection writing guideline

As a participant in the study ‘Exploring the psychological and academic adjustments of Chinese international students in the UK during the COVID-19, you are encouraged to provide much in-depth information with the researchers. What you reflect on and write up may constitute a major data source for the present study. Your writing will only be read by the researchers for academic use only. You can write about anything relevant to the themes of this study: your psychological and academic adjustments as a Chinese international student studying in the UK during the COVID-19. Specifically, your writing may include but not limited to the following guiding questions:

1. Your life trajectory in the quarantine and the COVID-19 in the UK;
2. Scenarios regarding your daily life, social life, and academic studies;
3. The stressors or difficulties or challenges you encounter during the COVID-19 in the UK;
4. Your response to these challenges;

5. Something else that helps you out with overcoming these difficulties;
6. Anything you want to share.

## Appendix III: data analysis and coding Scheme

| **Themes** | **Codes** | **Descriptors** | **Examples from the transcribed data** |
|---|---|---|---|
| COVID-Specific factors | Threat of infection | COVID-19 imposed threats to the health and well-being of Chinese international students | "I don't feel safe here…" (Participant 3, semi-structured interview #3)<br><br>"I have to wear masks everywhere, even in my flat…I think it is extremely risky and dangerous…" (Participant 6, stimulated recall interview) |
| | Reduced access to university facilities and resources | The transition to online teaching mode and the shutting-down of campuses led to the unavailability of many university resources | "I cannot access the photo scanning device in my department because it is closed temporarily…I need more real-life interactions with my supervisor, but COVID turns every teaching event online" (Participant 7, semi-structured interview #1) |
| COVID-enhanced factors | Anxiety | Anxiety exacerbated by social media use and pressure from parents | "They were very overacting to the virus in the UK and called me ten times a day, which didn't help at all but got me overly anxious" (Participant 2, self-reflection writing) |
| | Anti-Asian racism | Any terrible experiences related to Anti-Asian racism | "I was discriminated by a local teenager and he yelled that vulgar words to me and thought it was my fault [as a person from China] to bring COVID to the UK " |

| | | | |
|---|---|---|---|
| | | | (Participant 3, semi-structured interview #2) |
| | Hate incidents | Any hate incidents that these participants or people around them underwent | "my friend was unfortunately attacked by a group of local teenagers simply because he looked like a Chinese guy" (Participant 4, semi-structured interview#1) |
| | Online violence | Online discrimination against Chinese people in the UK | "the stigmatizing hashtags such as Chinese virus, Wuhan virus" (Participant 7, semi-structured interview #1) |
| Long-standing issues | Language barriers | The lack of oral English language proficiency | "I barely talk to the teacher and other classmates because I know my spoken English is not good enough to express my thoughts" (Participant 5, self-reflection writing) |
| | Academic differences | The differences between studying in China and studying in UK | "I find it hard to get used to the new learning style…here students are way more independent and they have clear study plans" (Participant 8, self-reflection writing) |
| Individual level strategies | previous experiences | Previous lockdown and any COVID-related experiences that could serve as resources for students to adjust to the living environment | "I had experienced physical and mental distress during the national lockdown in China…Life in the UK couldn't be worse" (Participant 5, semi-structured interview #2) |
| | Personal resilience | The ways in which participants exert their agency to overcome difficulties in new learning and living settings | "I am able to go through this period as an adult" (Participant 1, semi-structured interview #3) |

| | | | |
|---|---|---|---|
| Interpersonal level strategies | Monocultural network | Developing friendship with co-nationals | "I feel strongly connected and being understood by them [Chinese friends], and that is something my foreign friends can't give me" (Participant 2, self-reflection writing ) |
| | Bicultural network | Making friends with people other than Chinese to adjust to the host country | "I would like to chat with local friends, but they can give me some help" (Participant 3, stimulated recall interview ) |
| Institutional support | Life support | Any resources that help to protect students' physical and mental health in their daily life | The university started to provide online psychological counselling, COVID-19 helplines, personal tutor meetings, and COVID-19 hardship funds (Participant 8, memoing) |
| | Academic support | The efforts that universities made to support international students to overcome some academic difficulties | "such as making the essay deadline more flexible than before… boosting our confidence in online classes" (Participant 1, Semi-structured interview #3) |
